# Supplementary material for: Postpartum Depression after Maternal Isolation during the COVID-19 Pandemic: The MUMI-19 Study (Mothers Undergoing Mental Impact of COVID-19 Pandemic)
Source: J Clin Med. 2022 Sep 20;11(19):5504. doi: 10.3390/jcm11195504 (PMC9573123; doi:10.3390/jcm11195504)
Supplement: Supplementary file 1 [file jcm-11-05504-s001.zip › jcm-1836197-supplementary.pdf]

Supplementary Table S1 : Details of difficult obstetric history

	**Total**	**Lockdown group (ld group)**	**Post Lockdown Group (pld group)**
	n = 264	*n* = 129	*n* = 135
Pre-eclampsia	*n* = 8	*n* = 2	*n* = 6
Late term miscarriage	*n* = 1	*n* = 0	*n*= 1
Previous death in utero	*n* = 4	*n* = 2	*n* = 2
Neonatal death	*n* = 3	*n* = 0	*n* = 3
First child epilepsy	*n* = 1	*n* = 0	*n* = 1
Premature rupture of membranes	*n* = 1	*n* = 0	*n* = 1
Severe growth retardation	*n* = 5	*n* = 3	*n* = 2
Retro placental hematoma	*n* = 1	*n* = 0	*n* = 1
Delivery hemorrhage >1L	*n* = 2	*n* = 1	*n* = 1
Preterm delivery	*n* = 1	*n* = 1	*n* = 0
Threatened preterm delivery	*n* = 1	*n* = 1	*n* = 0
Toxoplasmosis seroconversion	*n* = 1	*n* 1	*n* = 0
Shoulder dystocia	*n* = 2	*n* = 1	*n* = 1
Rare genetic disease in siblings	*n* = 3	*n* = 1	*n* = 2
Medical termination of pregnancy	*n* = 2	*n* = 1	*n* = 1
Uterine rupture	*n* = 2	*n* = 0	*n* = 2

Supplementary Table S2: Subgroup analysis of secondary endpoints according to EPDS score at D30 and/or D60

	**Lockdown group (ld group)** *n* = 56				**Post Lockdown group (pld group)** *n* = 41				***p***
	Score<10.5	Score>10.5	Total	*p*	Score <10.5	Score >10.5	Total	*p*	
Feeling unable to care for the child	5/43 (11.6%)	7/13 (53.8%)	12/56 (21.4%)	0.003	2/28 (7.1%)	1/12 (8.3%)	3/40 (7.5%)	0.999	0.088
Spoiled start to motherhood	20/43 (46.5%)	7/13 (53.8%)	27/56 (48.2%)	0.756	0/28	1/12 (8.3%)	1/40 (2.5%)	0.300	<0.001
Apprehension of the return home by the partner									
D30	7/40 (17.5%)	2/11 (18.2%)	9/51 (17.6%)	0.999	1/29 (3.4%)	1/9 (11.1%)	2/38 (5.3%)	0.423	0.107
D60	6/26 (23.1%)	0/3	6/29 (20.7%)	0.999	0/19	0/4	0/23	0.999	0.028
Suffering expressed by partner	17/43 (39.5%)	9/13 (69.2%)	26/56 (46.4%)	0.111	2/28 (7.1%)	0/11	2/39 (5.1%)	0.999	<0.001
Breastfeeding									
proceeding D30	32/41 (78.0%)	11/11 (100%)	43/52 (82.7%)	0.177	27/29 (93.1%)	8/9 (88.9%)	35/38 (92.1%)	0.999	0.227
easy at D30	19/32 (59.4%)	7/11 (63.6%)	26/43 (60.5%)	0.999	22/27 (81.5%)	4/8 (50.0%)	26/35 (74.3%)	0.162	0.233
proceeding D60	21/27 (77.8%)	4/4 (100%)	25/31 (80.6%)	0.561	19/19 (100%)	3/4 (75.0%)	22/23 (95.7%)	0.174	0.218
easy at D60	11/21(52.4%)	2/4 (50.0%)	13/25 (52.0%)	0.999	17/19 (89.5%)	1/3 (33.3%)	18/22 (81.8%)	0.073	0.063
Psychological consultation desired	3/43 (7.0%)	3/13 (23.1%)	6/56(10.7%)	0.130	0/28	3/12 (25.0%)	3/40 (7.5%)	0.022	0.731
Early release	22/41 (53.7%)	1/12 (8.3%)	23/53 (43.4%)	0.007	6/28 (21.4%)	2/11 (18.2%)	8/39 (20.5%)	0.999	0.027

Supplementary Table S3: Subgroup analysis of EPDS score at D30 and/or D60 according to risk factors

	**Lockdown group (ld group)** *n* = 56		*p*	**Post Lockdown group (pld group)** *n* = 41		*p*
	Score <10.5 at D30 and/or D60 *n* = 43	Score >10.5 at D30 and/or D60 *n* = 13		Score <10.5 at D30 and/or D60 *n* = 29	Score >10.5 at D30 and/orD60 *n* = 12	
Age (years)	*n* = 43	*n* = 13	0.622	*n* = =28	*n* = 11	0.337
≤ 24	1/43 (2.3%)	1/13 (7.7%)		2/28 (7.1%)	0/11	
25-29	9/43 (20.9%)	4/13 (30.8%)		4/28 (14.3%)	4/11 (36.4%)	
30-34	20/43 (46.5%)	5/13 (38.5%)		12/28 (42.9%)	5/11 (45.4%)	
> 34	13/43 (30.2%)	3/13 (23.0%)		10/28 (35.7%)	2/11 (18.2%)	
Parity	*n* = 43	*n* = 13	0.001	*n* = 27	*n* = 11	0.471
nulliparity	17/43 (39.5%)	12/13 (92.3%)		10/27 (37.0%)	6 (54.5%)	
parity ≥1	26/43 (60.5%)	1/13 (7.7%)		17/27 (63.0%)	5 (45.5%)	
Presence of a history of violence in childhood	*n* = 43	*n* = 13	0.445	*n* = 28	*n* = 12	0.098
none	38/43 (88.4%)	11/13 (84.6%)		26/28 (92.8%)	9/12 (75.0%)	
psychological	2/43 (4.7%)	2/13 (15.4%)		0/28	2/12 (16.7%)	
physical	1/43 (2.2%)	0/13		1/28 (3.6%)	1/12 (8.3%)	
physical and psychological	2/43 (4.7%)	0/13		1/28 (3.6%)	0/12	
Presence of a medical history impacting on the pregnancy	7/43 (16.3%)	3/13 (23.1%)	0.682	3/27 (11.1%)	1/11 (9.1%)	0.999
Presence of a psychiatric history	3/43 (7.0%)	0/13	0.999	1/28 (3.6%)	3/12 (25.0%)	0.073
Presence of a family psychiatric history	7/43 (16.3%)	1/13 (7.7%)	0.665	6/28 (21.4%)	1/12 (8.3%)	0.652
Presence of a difficult obstetrical history	2/43 (4.7%)	0/13	0.999	2/28 (7.4%)	2/11 (18.2%)	0.564
Precarious situation	42/43 (97.7%)	11/13 (84.6%)	0.131	28/28 (100.0%)	12/12 (100.0%)	0.999
Homeless patient	43/43 (100.0%)	12/13 (92.3%)	0.232	9/27 (33.3%)	4/11 (36.4%)	0.999
Presence of marital difficulties	42/43 (97.7%)	13/13 (100.0%)	0.999	1/28 (3.6%)	0/12	0.999
Insufficient support for the patient	42/43 (97.7%)	13/13 (100.0%)	0.999	28/28 (100.0%)	12/12 (100.0%)	0.999

Existence of a migratory pathway for the patient	5/43 (11.6%)	1/13 (7.7%)	0.999	1/28 (3.6%)	0/12	0.999
Consumption of toxic substances	6/43 (14.0%)	3/13 (23.1%)	0.419	4/28 (14.3%)	0/12	0.267
Current unwanted pregnancy	38/43 (88.4%)	12/13 (92.3%)	0.999	27/28 (96.4%)	12/12 (100.0%)	0.999
Pregnancy difficult to obtain	6/39 (15.8%)	2/12 (16.7%)	0.999	2/28 (7.7%)	2/12 (16.7%)	0.577
Current pregnancy at risk	13/42 (31.0%)	3/11 (27.3%)	0.999	9/27 (33.3%)	4/11 (36.4%)	0.999
Presence of per-partum stress	16/42 (38.1%)	8/13 (61.5%)	0.202	13/28 (46.4%)	11/12 (91.7%)	0.012
Delivery	*n* = 43	*n* = 13	0.999	*n* = 27	*n* = 11	0.648
instrumental	31/43 (72.1%)	10/13 (76.9%)		21/27 (77.8%)	10/11 (90.9%)	
C-section	12/43 (27.9%)	3/13 (23.1%)		6/27 (22.2%)	1/11 (9.1%)	
Complications of childbirth	*n* = 43	*n* = 13	0.664	*n* = 27	*n* = 11	0.501
none	40/43 (93.1%)	12/13 (92.3%)		26/27 (96.3%)	10/11 (90.9%)	
Preterm birth	1/43 (2.3%)	1/13 (7.7%)		0/27	0/11	
Obstetrical anal sphincter injury (stage 3 or 4)	0/43	0/13		0/27	0/11	
severe delivery hemorrhage > 1000mL	1/43 (2.3%)	0/13		1 (3.7%)	1/11 (9.1%)	
uterine rupture	1/43 (2.3%)	0/13		0/27	0/11	
Traumatic experience of childbirth	5/42 (11.9%)	5/13 (38.5%)	0.045	0/27	2/12 (16.7%)	0.085
Absence of partner at delivery	*n* = 43	*n* = 13	0.999	*n* = 28	*n* = 12	0.300
present	6/43 (14.0%)	1/13 (7.7%)		0/28	0/12	
unwanted absence	35/43 (81.4%)	12/13 (92.3%)		28/28 (100.0%)	11/12 (91.7%)	
desired absence	2/43 (4.7%)	0/13		0/28	1/12 (8.3%)	
Separation mother/child	3/43 (7.0%)	1/13 (7.7%)	0.999	0/28	0/12	0.999
Length of stay >3 days	18/37 (48.6%)	9/12 (75.0%)	0.332	14/26(53.8%)	2/10 (20.0%)	0.133
Breastfeeding	*n* = 43	*n* = 13	0.221	*n* = 28	*n* = 12	0.039
artificial	21/43 (48.8%)	8/13 (61.5%)		23/28 (82.1%)	5/12 (41.7%)	
easy breastfeeding	13/43 (30.2%)	5/13 (39.5%)		4/28 (14.3%)	5/12 (41.7%)	
complicated breastfeeding	9/43 (20.9%)	0/13		1/28 (3.6%)	2/12 (16.6%)	
